# Classical Soybean (*Glycine max (L.) Merr*) Symbionts, *Sinorhizobium fredii* USDA191 and *Bradyrhizobium diazoefficiens* USDA110, Reveal Contrasting Symbiotic Phenotype on Pigeon Pea (*Cajanus cajan* (L.) Millsp)

**DOI:** 10.3390/ijms20051091

**Published:** 2019-03-03

**Authors:** Alaa A. Alaswad, Nathan W. Oehrle, Hari B. Krishnan

**Affiliations:** 1 Plant Science Division, University of Missouri, Columbia, MO 65211, USA; alaaalaswad@mail.missouri.edu; 2 King Abdul Aziz University, 21589 Jeddah, Saudi Arabia; 3 Plant Genetics Research Unit, USDA-Agricultural Research Service, Columbia, MO 65211, USA; Nathan.Oehrle@ARS.USDA.GOV

**Keywords:** Pigeon pea, nodulation, biological nitrogen fixation, type 3 secretion system, *Bradyrhizobium diazoefficiens*, *Sinorhizobium fredii*

## Abstract

Pigeon pea (*Cajanus cajan (L.)* Millspaugh) is cultivated widely in semiarid agricultural regions in over 90 countries around the world. This important legume can enter into symbiotic associations with a wide range of rhizobia including *Bradyrhizobium* and fast-growing rhizobia. In comparison with other major legumes such as soybean and common bean, only limited information is available on the symbiotic interaction of pigeon pea with rhizobia. In this study, we investigated the ability of two classical soybean symbionts—*S. fredii* USDA191 and *B. diazoefficiens* USDA110—and their type 3 secretion system (T3SS) mutants, to nodulate pigeon pea. Both *S. fredii* USDA191 and a T3SS mutant *S. fredii* RCB26 formed nitrogen-fixing nodules on pigeon pea. Inoculation of pigeon pea roots with *B. diazoefficiens* USDA110 and *B. diazoefficiens* Δ136 (a T3SS mutant) resulted in the formation of Fix− and Fix+ nodules, respectively. Light and transmission electron microscopy of Fix- nodules initiated by *B. diazoefficiens* USDA110 revealed the complete absence of rhizobia within these nodules. In contrast, Fix+ nodules formed by *B. diazoefficiens* Δ136 revealed a central region that was completely filled with rhizobia. Ultrastructural investigation revealed the presence of numerous bacteroids surrounded by peribacteroid membranes in the infected cells. Analysis of nodule proteins by one- and two-dimensional gel electrophoresis revealed that leghemoglobin was absent in *B. diazoefficiens* USDA110 nodules, while it was abundantly present in *B. diazoefficiens* Δ136 nodules. Results of competitive nodulation assays indicated that *B. diazoefficiens* Δ136 had greater competitiveness for nodulation on pigeon pea than did the wild type strain. Our results suggest that this T3SS mutant of *B. diazoefficiens*, due to its greater competitiveness and ability to form Fix+ nodules, could be exploited as a potential inoculant to boost pigeon pea productivity.

## 1. Introduction

Symbiotic nitrogen fixation is a great example of an association between plants and microorganisms that significantly enhances crop production. It results in the formation of new organs called nodules on either the roots or stems of legume plants. In the soil, legume roots are exposed to diverse microorganisms and legumes and rhizobia must recognize each other by a series of specific signal exchanges to establish symbiosis. Flavonoids secreted by legume roots are key signal molecules that are perceived by the rhizobia. Diverse flavonoids are elaborated by different legumes and are recognized by specific rhizobial species, resulting in the first level of symbiosis specificity [1].

The perception of the root signals is mediated by the NodD protein, a transcriptional regulator of nodulation (*nod*) genes in the rhizobia. Specific flavonoids interact with NodD, leading to the activation of *nod* genes. Some of *nod* genes are involved in the production of a strain-specific nodulation signaling molecule called Nod factor (NF) which activates the host signaling pathway [2]. The core structure of NF is encoded by *nod*ABC genes and it is believed to be conserved across all rhizobia species, except for two Aeschynomene-infective species [3]. In addition to NF, exopolysaccharides (EPS) also plays a vital role in the establishment of effective symbiosis in many rhizobial–legume models [4,5]. A plant receptor protein interacting with rhizobial EPS has been recently identified [6]. By employing rhizobial and plant mutants it has been demonstrated that bacterial infection of legume roots is regulated by receptor-mediated recognition of Nod factor and EPS signals [6]. The presence of the compatible rhizobia and their corresponding NF is usually sufficient to trigger nodule formation [7]. However, successful symbiosis can be affected by several factors, linked to both the environment and the symbiotic partners, which can either promote or prevent nodulation [8]. Rhizobia enter legume root hairs via the infection thread, a tubular structure that facilitates rhizobial entrance into host root cortical cells [9]. Within the cortical cells, the rhizobia released from the infection thread differentiate into specialized units called bacteroids. The bacteroids are surrounded by peribacteroid membranes of plant origin and facilitate the conversion of atmospheric nitrogen to ammonia [10]. The fixed nitrogen promotes the growth and development of the legume host.

The type 3 secretion system (T3SS) plays an important role in the pathogenicity of many Gram-negative bacteria that infect humans, animals and plants [11]. By employing the T3SS, pathogens transport effector proteins into the eukaryotic host cells that aid in the development of disease [11,12]. Even though the T3SS initially was thought to be unique to pathogenic bacteria, its presence in several symbiotic rhizobia is now well established [13,14]. The genome sequence of many pathogenic and symbiotic bacteria has revealed the presence of at least six secreting systems (T1SS–T6SS) based on their secretion mechanisms [15]. Many of the rhizobia possess T3SS, T4SS, and/or T6SS to deliver their secreted proteins into the cytoplasm of the host cell [16]. The proteins secreted by rhizobia via T3SS are known as nodulation outer proteins (Nops) [17]. These proteins influence nodulation either by promoting or suppressing nodule development depending on the specific host. A functional T3SS has been reported in *Sinorhizobium fredii* USDA257 [18], *S. fredii* USDA191, *S. fredii* HH103 [19], *Rhizobium* strain NGR234 [20], *Bradyrhizobium diazoefficiens* USDA110 [21], *B. elkanii* [22], and *Mesorhizobium loti* MAFF303999 [23]. Our lab has been investigating cultivar-specific nodulation of soybean by a fast-growing rhizobium, *S. fredii* USDA257. This strain nodulates primitive soybean cultivars such as Peking, but not agronomically improved North American cultivars [24,25,26,27]. The ability of *S. fredii* USDA257 to nodulate soybean in a cultivar-specific manner is mediated by the Nops that are secreted by T3SS [13]. T3SS mutants of both *S. fredii* USDA257 and *B. diazoefficiens* USDA110 have been reported to either promote or inhibit nodulation in a host specific manner [18,21].

Pigeon pea is an important legume grown in semiarid agricultural regions in over 90 countries around the world. It is cultivated predominately in Asia, Africa, Latin America and Australia. Compared with other grain legumes, pigeon pea ranks only sixth in area and production [28]. This legume is protein-rich, with an average seed protein content of 24%, but it can also be as high as 31% of the overall seed dry weight [29]. Due to its nutritional quality and availability, it is abundantly consumed in developing countries to meet people’s nutritional needs [29]. Pigeon pea benefits from symbiotic nitrogen fixation, obtaining approximately 77–90% of the N that is required for its physiological development [30,31,32]. A wide range of rhizobia including *Bradyrhizobium* (cowpea group) and fast-growing rhizobia have been reported to nodulate pigeon pea. The molecular diversity of pigeon pea-nodulating rhizobia has been investigated for the purpose of classifying these rhizobia and evaluating their symbiotic effectiveness. Matrix-assisted laser desorption/ionization time-of-flight (MALDI-TOF) mass spectrometry (MS) and DNA sequencing have been utilized to identify rhizobia from pigeon pea nodules in the fields of Côte d’Ivoire [33]. This study identified two major clades of bradyrhizobia, one of which belongs to the *Bradyrhizobium elkanii* super clade. Several of the isolates exhibited superior symbiotic efficiencies highlighting their potential as a pigeon pea inoculant [33]. A recent study isolated 116 nitrogen-fixing rhizobial strains from root nodules of pigeon pea grown in Ethiopia [34]. Based on several phenotypic traits, these isolates were found to be related either to *Bradyrhizobium japonicum* (HAMBI 2314T) or *Bradyrhizobium elkanii* (LMG 6164). Evaluation of the symbiotic effectiveness of these isolates revealed that most of them were found to be effective nitrogen fixers [34]. Though some progress has been made to identify nitrogen-fixing rhizobial isolates [33,34,35], further research is required to identify and characterize pigeon pea-nodulating rhizobia that exhibit greater symbiotic effectiveness even under adverse environmental conditions. During the course of investigating the nodulation phenotype of T3SS mutants, we observed T3SS mutants of *S. fredii* USDA191 and *B. diazoefficiens* USDA110 that revealed contrasting nodulation phenotype on pigeon pea. In this report, we have conducted a study on the nodulation response of pigeon pea to two classical soybean symbionts and their T3SS mutants.

## 2. Results

### 2.1. Nodulation Outer Proteins (NOPs) of Sinorhizobium fredii USDA191, Bradyrhizobium diazoefficiens USDA110, and their T3SS Mutants

**Figure 1:**
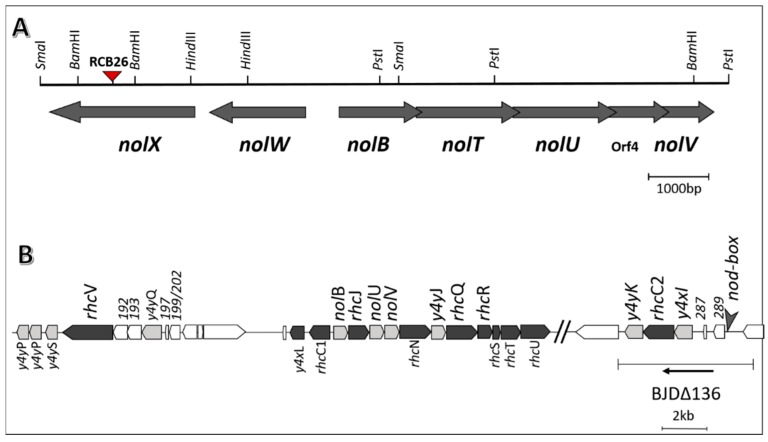
Physical and genetic map of T3SS locus of *S. fredii* USDA191 (**A**) [39] and *B. diazoefficiens* USDA110 (**B**) [21]. The position of mini-Mu insertion in NopX (NolX) of USDA191 is shown with a red triangle (**A**). The DNA region deleted in *B. diazoefficiens* Δ136 is shown (**B**).

**Figure 2:**
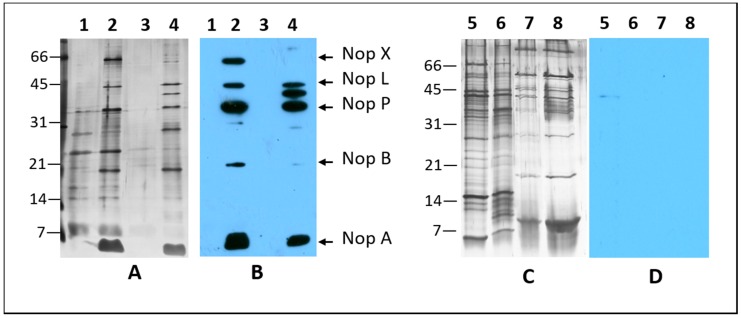
Extracellular protein profile and immunoblot analysis of Nops. Extracellular proteins produced by *S. fredii* USDA191 (lanes 1 and 2), RCB26 (lanes 3 and 4), *B. diazoefficens* USDA110 (lanes 5 and 6), and Δ136 (lanes 7 and 8) were resolved by 15% SDS-PAGE and silver stained (**A**,**C**) or transferred to nitrocellulose membrane for immunoblot analysis (**B**,**D**). Nops were detected with a cocktail of antibodies raised against individual Nop proteins. Odd and even number lanes contain proteins isolated from cells grown in the absence or presence of 1 µM apigenin or genistein, respectively. The size of the molecular weight markers and the identity of Nops are shown at the sides of the figure.

*S. fredii* USDA191 and *B. diazoefficiens* USDA110, nitrogen-fixing symbionts of soybean, elaborate extracellular proteins when grown in presence of *nod* gene inducers such as apigenin and genistein [13,21]. The T3SS mutant line *S. fredii* RCB26 contains a mini-Mu insertion in the *nopX* gene while *B. diazoefficiens* Δ136 lacks a functional *ttsI* gene and several neighboring genes (Figure 1). To examine if the T3SS mutants of these two soybean symbionts were defective in Nops production, we first isolated the extracellular proteins produced by these rhizobia in presence of apigenin or genistein (Figure 2). *S. fredii* USDA191, when grown in presence of apigenin, produced several prominent proteins that were not present in uninduced cultures. *S. fredii* USDA191 T3SS mutant line RCB26 also produced a similar set of Nops with the exception of a 64 kDa protein. Unlike *S. fredii* USDA191, induction of B. *diazoefficiens* with apigenin did not result in the production of any prominent extracellular proteins (data not shown) indicating that apigenin may not be a strong inducer of USDA110 nodulation genes. Subsequently, we used genistein for the induction of Nops. In the absence of the inducer, *B. diazoefficiens* USDA110 produced several extracellular proteins whose molecular weights ranged from 66 kDa to less than 7 kDa (Figure 2). Flagellins, the major structural proteins of the flagella, have been identified as the most abundant proteins secreted by *B. diazoefficiens* [36]. *B. diazoefficiens* flagellins migrate as two bands on SDS-PAGE gels with apparent molecular masses of 65 and 33 kDa, respectively [37]. Similar-sized proteins were also observed in our study (Figure 2). Addition of genistein to growth media resulted in the production several new proteins some of which had molecular weights lower than 10 kDa. Earlier it has been shown genistein induction of *B. diazoefficiens* USDA110 results in the production of several Nops including NopB, NopH, NopT, GunA2, and PgI [36,38]. Interestingly, the T3SS mutant line Δ136 produced large amounts of exopolysaccharides which interfered with the isolation of extracellular proteins and subsequent migration of these proteins on SDS-PAGE gels. The extracellular proteins produced by the T3SS mutant line Δ136 in the absence or presence of genistein was mostly similar to each other (Figure 2). Interestingly, some of the low molecular weight proteins which are present in genistein-induced cultures of USDA110 were not present in the T3SS mutant line Δ136. Our observation is consistent with an earlier report where genistein-inducible extracellular proteins were not detected in a mutant strain where the *tts* gene cluster was removed [36].

Identification of Nops in the extracellular protein fraction was examined by immunoblot analysis using a cocktail of *S. fredii* Nop specific antibodies [18]. This analysis confirmed the accumulation of NopX, NopL, NopP, NopB, and NopA in the induced cultures of *S. fredii* USDA191. In contrast, T3SS mutant line RCB26 failed to accumulate NopX, instead a protein with apparent molecular weight 40 kDa was detected (Figure 2). *S. fredii* Nop-specific antibodies; however, failed to react with any extracellular proteins produced by *B. diazoefficiens* USDA110 and its T3SS mutant line Δ136. This observation is not surprising since we employed a cocktail of antibodies raised against *S. fredii* NopX, NopL, NopP, NopB, and NopA, some of which are not produced by *B. diazoefficiens* USDA110 [36,38]. Another reason why we were unable to detect the accumulation of these proteins could be related to low abundance of these proteins in the extracellular protein fractions. Furthermore, the Nop proteins of *S. fredii* and *B. diazoefficiens* show only limited amino acid identity and thus antibodies raised against *S. fredii* and may not recognize the similar protein from *B. diazoefficiens*.

### 2.2. Sinorhizobium fredii USDA191 Exhibits Nitrogen Fixing Nodules While Bradyrhizobium diazoefficiens USDA110 Forms Ineffective Nodules on Pigeon Pea

**Figure 3:**
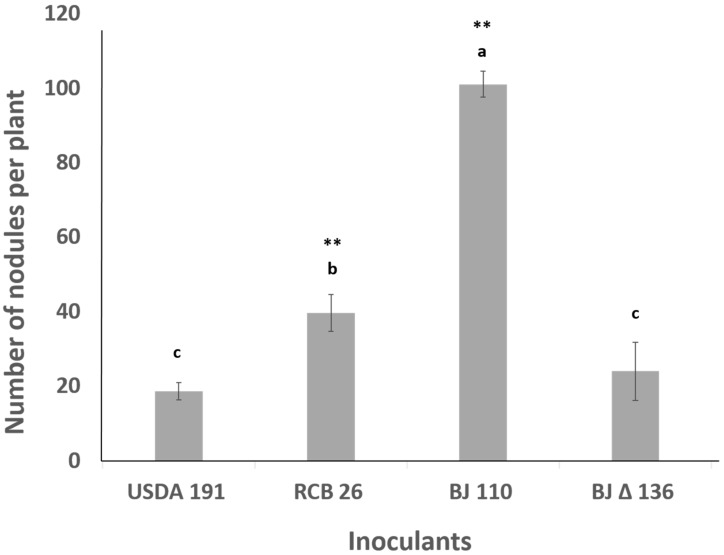
Nodulation of pigeon pea by *B. diazoefficiens* USDA110, *S. fredii* USDA191, and T3SS mutants. The bars represent the total number of nodules per plant and standard errors are indicated above bars. Bars with same letter are not significantly different. Values are presented as mean ± SD (*n* = 12). Double asterisks (**) indicate significance at the *p* < 0.01 level. Letters indicate the result of Tukey–Kramer test for significant ANOVA results.

*S. fredii* USDA191 and *B. diazoefficiens* USDA110 have been documented to produce nitrogen-fixing nodules on soybean [19,21]. We examined the response of these two soybean symbionts on pigeon pea nodulation. Both of these soybean symbionts revealed determinate nodules on pigeon pea (Figure S1). Interestingly, *B. diazoefficiens* USDA110 produced many small white nodules in contrast to *S. fredii* USDA191 which produced pinkish nodules that were noticeably larger in size and more sequestered (Figure S1). We also examined the nodulation phenotype of pigeon pea when inoculated with T3SS mutants of *S. fredii* USDA191 and *B. diazoefficiens* USDA110. In contrast to the parental strain, the *B. diazoefficiens* T3SS mutant Δ136 produced nodules (Figure S1). However, the number of nodules produced by this T3SS mutant was significantly lower than that induced by the wild type (Figure 3). Inoculation of pigeon pea roots with a *S. fredii* USDA191 T3SS mutant line RCB26 (NolX mutant), did not drastically alter the nodulation response. Like the wild type, RCB26 also produced nodules. The number of nodules produced by RCB26 on pigeon pea was consistently higher than the wild type (Figure 3). To verify that indeed these nodules produced by both the parental strains and their respective T3SS mutants were effective at nitrogen fixation, acetylene reduction assays were performed on each group using intact, whole nodules. Results showed that *S. fredii* USDA191 (1.18 nmol C_2_H_4_ hr^−1^ mg^−1^ nodule ± 0.06), *S. fredii* USDA RCB26 (0.65 nmol C_2_H_4_ hr^−1^ mg^−1^ nodule ± 0.04), and *B. diazoefficiens* Δ136 (0.47 nmol C_2_H_4_ hr^−1^ mg^−1^ nodule ± 0.02) were effective nitrogen-fixing nodules. However, pigeon pea inoculated with *B. diazoefficiens* USDA110, while having a significantly higher number of nodules than the others (Figure 3), had zero acetylene reduction activity. Pigeon pea plants inoculated with *B. diazoefficiens* USDA110 had significantly lower shoot fresh weight (Figure S2) and smaller-sized nodules (Figure S3) when compared with other inoculants.

### 2.3. Anatomy of Pigeon Pea Nodules Induced by Sinorhizobium fredii USDA191 and Bradyrhizobium diazoefficiens USDA110

**Figure 4:**
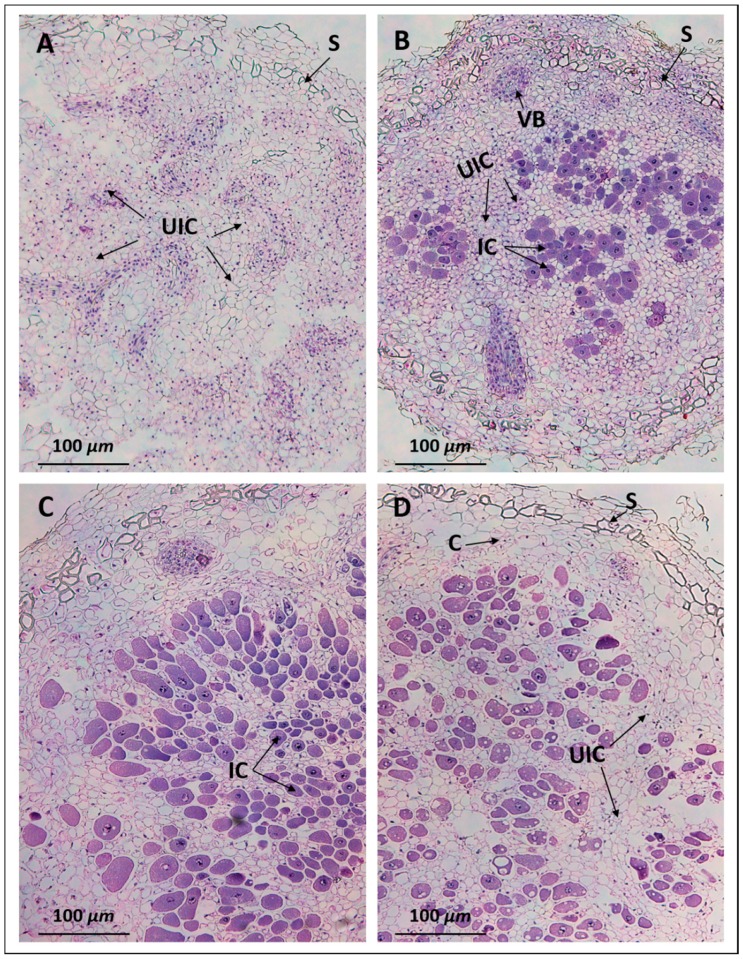
Light micrographs of nodules. Pigeon pea nodules collected at 30 days after inoculation (DAI) were embedded in paraffin. Nodule sections were stained with hematoxylin and eosin. Note the nodules initiated by *B. diazoefficiens* USDA110 (**A**) contain only uninfected cells, while those inoculated with *B. diazoefficiens* Δ136 (**B**), *S. fredii* USDA191 (**C**), and *S. fredii* USDA RCB26 (**D**) revealed a distinct central infection zone. VB, vascular bundle; S, scleroid layer; UIC, uninfected cell; C, cortex.

Light microscopic examination of 30 days after inoculation (DAI) pigeon pea nodules initiated by *B. diazoefficiens* USDA110 revealed actively dividing cells that contained prominent nuclei (Figure 4A). Strikingly, these cells did not contain any rhizobia. However, the central mass of cells was surrounded by a scleroid layer. In contrast, an examination of the nodules formed by the T3SS mutant line Δ136, revealed anatomical features that were typical of well-defined functional nodules. The outermost layer (epidermis) and the cortex were separated by a scleroid layer (Figure 4B). Several vascular bundles were seen in the outer cortex region. The cortex, which was mainly made up of parenchymatous cells, contained both infected and uninfected cells. The infected cells were easily distinguishable from the uninfected cells due to their prominent staining with hematoxylin and eosin (Figure 4B). An examination of the nodules produced by USDA191 and its T3SS mutant line RCB26, revealed similar structural features as observed in Δ136 nodules (Figure 4C,D). However, a higher proportion of parenchymatous cells were infected by *S. fredii* when compared to that of *B. diazoefficiens* Δ136 (compare Figure 4B to Figure 4C with Figure 4D).

### 2.4. Ultrastructural Observation of Pigeon Pea Nodules Produced by Sinorhizobium fredii USDA191 and Bradyrhizobium diazoefficiens USDA110 and their T3SS Mutants

**Figure 5:**
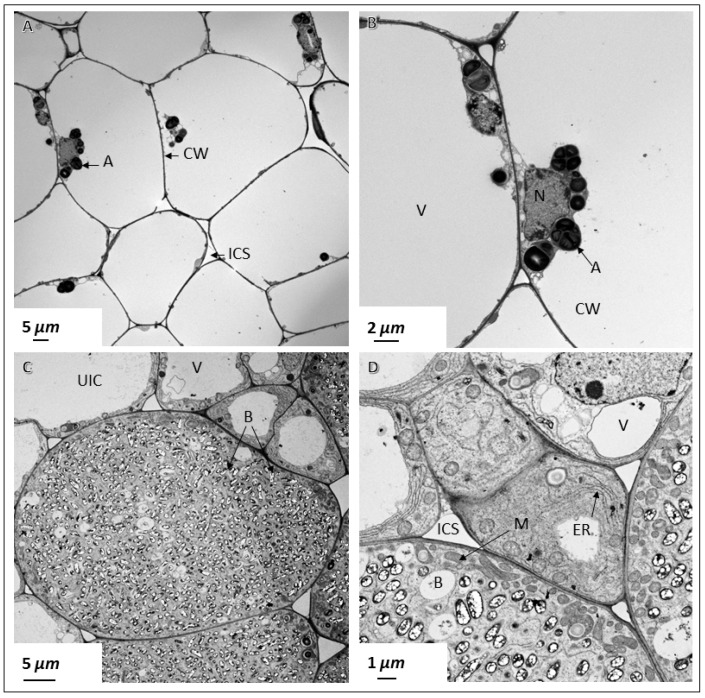
Transmission electron micrographs of pigeon pea nodules. Thin sections of 30 DAI pigeon pea nodules were examined by transmission electron microscopy. Nodules elicited by *B. diazoefficiens* USDA110 contain no rhizobia inside the cells (**A**,**B**). These cells contain prominent vacuoles (V) that take up most of the cellular space (**A**,**B**). *B. diazoefficiens* Δ136-infected cells contain large number of bacteroids (**C**,**D**). CW, cell wall; ICS, intercellular space; M, mitochondria; ER, endoplasmic reticulum; N, nucleus; PBM, peribacteroid membrane; B, bacteroid; A, amyloplast; V, vacuole; UIC, uninfected cell.

**Figure 6:**
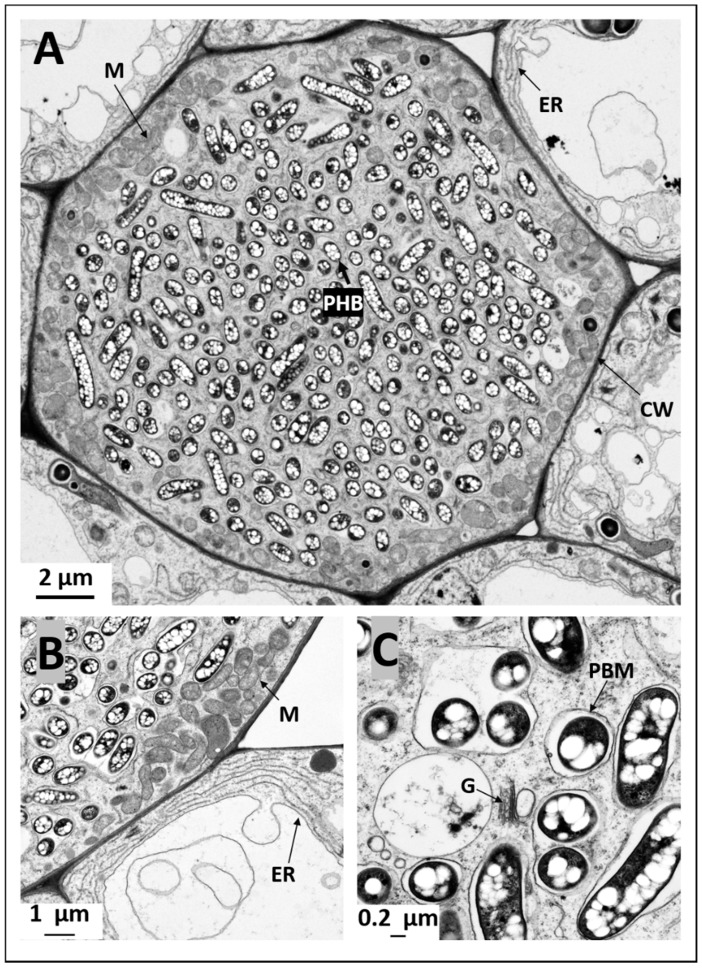
Transmission electron micrographs of pigeon pea nodules inoculated with *S. fredii* USDA191. Thin sections of pigeon pea nodules at 30 DAI were examined by transmission electron microscopy. The infected cells are filled with bacteroids (**A**). Note the presence of numerous mitochondria in the infected cells and prominent poly-β-hydroxybutyrate granules within the bacteroids (**B**,**C**). Extensive endoplasmic reticulum network is evident in uninfected cells (**A**,**B**). CW, cell wall; M, mitochondria; ER, endoplasmic reticulum; PBM, peribacteroid membrane; G, Golgi body; PHB, poly-β-hydroxybutyrate.

To investigate whether nodules produced by *Sinorhizobium fredii* USDA191 and *Bradyrhizobium diazoefficiens* USDA110 and their T3SS mutants exhibited any ultrastructural differences we examined thin sections of pigeon pea nodules at 30 DAI by transmission electron microscopy. TEM examination of *B. diazoefficiens* USDA110 nodules confirmed our light microscopic observation that demonstrated the complete absence of rhizobia in the cortical cells (Figure 5A,B). These cells were mostly empty and contained few organelles that were adhered to the thin cell walls (Figure 5A,B). In some cells, the nucleus was prominent and was often seen surrounded by starch grains (Figure 5B). In contrast, an examination of thin sections of *B. diazoefficiens* Δ136 nodules revealed cortical cells that were densely packed with rhizobia (Figure 5C). At this developmental stage, rhizobia had differentiated into nitrogen-fixing bacteroids and were surrounded by peribacteroid membranes (symbisome). Usually, individual symbiosomes contained one to three bacteroids inside them. These bacteroids contained prominent poly-β-hydroxybutyrate granules. Numerous mitochondria were prominently seen near the cell walls of these infected cells (Figure 5C). An examination of the uninfected cells surrounding the infected cells revealed the presence of an extensive endoplasmic reticulum network (Figure 5D). These cells also contained numerous mitochondria but unlike those found in the infected cells, these mitochondria appeared to be spherical. Prominent vacuoles were also observed in the uninfected cells (Figure 5D). Transmission electron microscopic examination of pigeon pea nodules initiated by *S. fredii* USDA191 showed cells that were completely occupied by rhizobia (Figure 6A). At this stage of nodule development, the bacteroids enclosed inside symbiosomes appeared either elongated or spherical in shape. Regardless of the shape, the bacteroids contained numerous poly-β-hydroxy-butyrate granules (Figure 6A–C).

**Figure 7:**
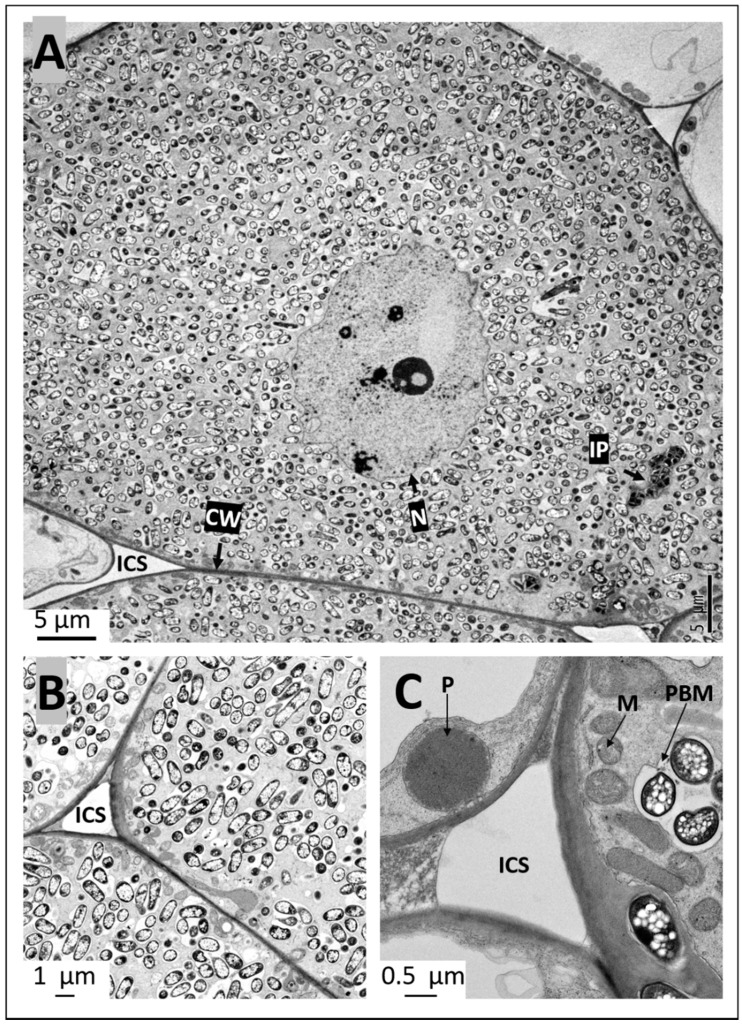
Transmission electron micrographs of pigeon pea nodules inoculated with *S. fredii* USDA RCB26. Thin sections of pigeon pea nodules at 30 DAI were examined by transmission electron microscopy. The infected cells are filled with bacteroids (**A**,**B**). Note the presence of infection pocket (IP) and prominent nucleus (**A**). Prominent mitochondria were seen in the infected cells (**C**) and peroxisomes were evident in uninfected cells (**C**). CW, cell wall; ICS, intercellular space; M, mitochondria; N, nucleus; PBM, peribacteroid membrane; P, peroxisome.

High magnification observation of the symbiosomes revealed the presence of varying number of bacteroids inside them. Occasionally, the symbiosomes appeared as sac-like structures that contained remnants of degenerating bacteroids (Figure 6C). Golgi apparatus were also commonly found in the infected cells (Figure 6C). Strikingly, numerous mitochondria were seen all around the outer region of the infected cells close to the cell walls (Figure 6A,B). Even though mitochondria were commonly encountered in the uninfected cells, their numbers were much lower compared with the number found in the infected cells. Extensive endoplasmic reticulum network and prominent vacuoles were the most conspicuous features observed in the uninfected cells (Figure 6A,B). Similar ultrastructural features were also observed in pigeon pea nodules initiated by *S. fredii* USDA191 T3SS mutant line *S. fredii* RCB26 (Figure 7A–C). Even within bacteroid-filled cortical cells, occasionally the presence of infection threads/infection pockets were detected (Figure 7A). Prominent peroxisomes were commonly observed in uninfected cells of *S. fredii* RCB26 nodules (Figure 7C).

### 2.5. Two-Dimensional Gel Analysis of Nodule Proteins

**Figure 8:**
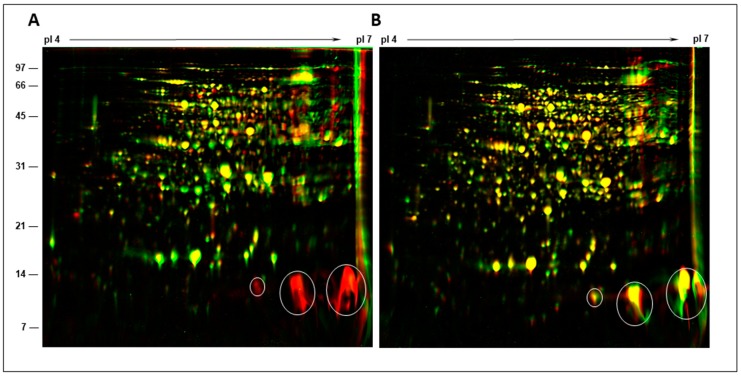
Comparison of nodule cytosol proteins by 2-D gel electrophoresis. Cytosol proteins isolated from 30-day-old pigeon pea nodules initiated by *B. diazoefficiens* USDA110 (green color), *B. diazoefficiens* Δ136 (red color) (**A**), *S. fredii* USDA191 (green color), and *S. fredii* RCB26 (red color) (**B**) were separated by isoelectric focusing (pH 4 to 7) followed by SDS-PAGE on a 15% gel. The proteins were visualized by staining the gel with Colloidal Comassie Blue G-250. These gels were scanned and proteins from wild type were assigned green color and those from T3SS mutants were given red color. The gels were overlaid using Delta2D software to detect protein differences. Yellow represents equal amounts of protein, red indicates lower abundance of a specific protein in wild type formed nodule and green color points to an increase in the abundance of protein in the T3SS mutant induced nodules. Leghemoglobulin protein spots are circled. The sizes of molecular weight markers in kDa are shown on the left side of the figure.

Several plant genes called ‘nodulins’ are specifically expressed in legume nodules [31,32]. These proteins play an important role in the infection process, nodule morphogenesis, nutrient transport, and nitrogen fixation [40]. Our morphological observations and results obtained from anatomical studies clearly demonstrated that *B. diazoefficiens* USDA110 initiated nodules on pigeon pea were incapable of nitrogen fixation since they were completely devoid of rhizobia. This observation prompted us to investigate whether these nodules revealed any detectable protein changes when compared with nitrogen-fixing pigeon pea nodules. For this purpose, we isolated cytosol proteins from pigeon pea nodules elicited by *S. fredii* USDA191, *B. diazoefficiens* USDA110, and their T3SS mutants and resolved them by high-resolution 2-D gel electrophoresis (Figure 8). Cytosol proteins isolated from pigeon pea nodules 30 DAI elicited by *B. diazoefficiens* USDA110 and Δ136 were resolved into several hundred protein spots with distinct isoelectric points. To visualize the differences in the protein profiles we overlaid the 2-D gel images of *B. diazoefficiens* USDA110 and Δ136 nodules using Delta2D software (Figure 8A).

This analysis clearly revealed that *B. diazoefficiens* USDA110 nodules were lacking some abundant protein spots with apparent molecular weight of 14 kDa. Though changes in the abundance of a few other protein spots were detected, the overall protein profile was not drastically different between *B. diazoefficiens* USDA110 and Δ136 nodules (Figure S4 and Table S1). We also compared the 2-D protein profiles of *S. fredii* USDA191 and RCB26 nodules (Figure 8B). In contrast to the *B. diazoefficiens* USDA110 and Δ136 nodules, the protein profiles of *S. fredii* USDA191 and RCB26 nodules were strikingly similar and revealed only marginal variation in the intensity of a few protein spots (Figure S5 and Table S2).

### 2.6. Leghemoglobin and Nitrogenase Are Absent in Pigeon Pea Nodules Produced by Bradyrhizobium diazoefficiens USDA110

**Figure 9:**
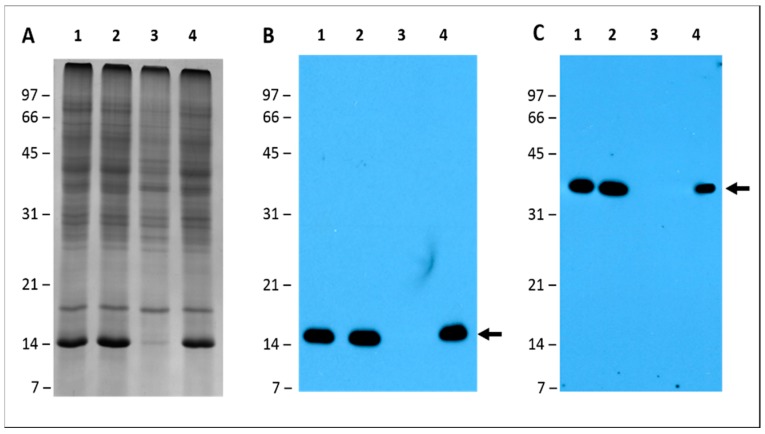
Leghemoglobin and nitrogenase detection by immunoblot analysis. Three identical 15% SDS-PAGE gels were used to resolve pigeon pea total nodule proteins. One gel was visualized by staining with Coomassie Brilliant Blue (**A**) and the other two gels were electrophoretically transferred to a nitrocellulose membrane and probed with either the leghemoglobin antibody (**B**) or nitrogenase antibody (**C**). Immunoreactive proteins were detected using anti-rabbit IgG−horseradish peroxidase conjugate followed by chemiluminescent detection. The arrows point to the proteins reacting to the leghemoglobin and nitrogenase antibody, respectively. Lanes: 1, *S. fredii* USDA191 nodules; 2, *S. fredii* USDA RCB26 nodules; 3, *B. diazoefficiens* USDA110 nodules; 4, *B. diazoefficiens* Δ136 nodules. Molecular weight markers are shown on the left and designated in kDa.

In legumes, an important nodule protein involved in the symbiotic nitrogen fixation process is leghemoglobin. This protein has been only detected in the infected tissue of root or stem nodules of actively nitrogen fixing plants [41]. It functions as an oxygen scavenger and facilitates its diffusion to the nitrogen-fixing microbial symbionts in nodules. This protein is present at a concentration to maintain a specific level of O_2_ in the cytosol of host cells, thereby permitting a suitable supply of ATP for nitrogen-fixation, but at the same time avoiding nitrogenase inactivation by oxygen [42]. The molecular weight of leghemoglobin ranges from 15 to 17 kDa and represents one of the most abundant proteins in legume nodules. Our 2-D gel analysis of nodule cytosol proteins revealed that *B. diazoefficiens* USDA110 produced pigeon pea nodules that were missing abundant protein spots with apparent molecular weight of 14 kDa. As a consequence of their size and abundance we suspected that these proteins may represent members of leghemoglobin. To confirm their identity, we performed Western blot analysis using an antibody raised against leghemoglobin. The antibody specifically recognized a 14 kDa protein present in the total protein extracts from nodules produced by *S. fredii* USDA191, RCB26, and *B. diazoefficiens* Δ136 nodules (Figure 9B). However, the leghemoglobin antibody failed to react against any protein confirming the absence of this protein in *B. diazoefficiens* USDA110 nodule extract. In addition to leghemoglobin, we also investigated the presence of nitrogenase: a protein responsible for the reduction of N_2_ to NH_3_. Antibodies raised against nitrogenase reacted against a 40 kDa protein present in the total protein extracts from nodules produced by *S. fredii* USDA191, RCB26, and *B. diazoefficiens* Δ136 nodules (Figure 9C). However, the antibody failed to react with any protein from *B. diazoefficiens* USDA110 nodule extract.

### 2.7. Coinoculation with Bradyrhizobium diazoefficiens T3SS Mutant Drastically Lowers the Number of Nodules Formed by the Wild Type Strain

**Figure 10:**
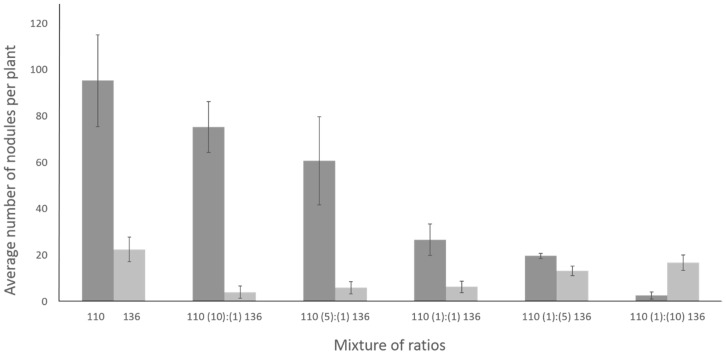
Competitive ability of wild type and T3SS mutants of *B. diazoefficiens* USDA110 to form nodules on pigeon pea. Pigeon pea roots where inoculated individually with *B. diazoefficiens* USDA110 or *B. diazoefficiens* Δ136 or inoculation mixtures that contained different proportions of wild type and T3SS mutant. Nodules were counted after 30 DAI and sorted into two groups: small, white interior nodules (Fix−) were grouped as those initiated by *B. diazoefficiens* USDA110, while relatively large, pink interior nodules (Fix+) were counted as those formed by *B. diazoefficiens* Δ136. Note the presence of T3SS mutant significantly lowers the number of nodules formed by *B. diazoefficiens* USDA110 on pigeon pea.

The impact of T3SS mutation on competitive nodulation on pigeon pea was examined by coinoculation with the wild type strain and T3SS mutant. The competitive ability of these strains of rhizobia to nodulate pigeon pea was measured by inoculating the roots with inoculant mixtures that contained various proportion of *B. diazoefficiens USDA110* and *B. diazoefficiens* Δ136 ranging from 10:1 to 1:10, respectively (Figure 10). As shown earlier, individual inoculation of *B. diazoefficiens USDA110* and *B. diazoefficiens* Δ136 on pigeon pea resulted in approximately 100 and 25 nodules, respectively (Figure 3). When these rhizobia were inoculated at 1:1 ratio, there was significant drop in the overall nodule numbers. Since *B. diazoefficiens USDA110* induced nodules were Fix- and had a white interior, it was easy to monitor the nodule occupancy by these two strains. Strikingly, the presence of T3SS mutant in the inoculation mixture lowered the ability of *B. diazoefficiens USDA110* to form nodules on pigeon pea. This negative effect was evident, albeit a lesser extent, even when the T3SS mutant was added at 10- and 5-fold lower concentration than the wildtype. Reciprocally, the presence of wild type strain in the inoculant mixture also lowered the ability of T3SS mutant to form nodules on pigeon pea. Interestingly, the negative effect of wild type strain to suppress the T3SS mutant nodule number was alleviated by increasing the concentration of the T3SS mutant in the inoculant mixture.

## 3. Discussion

In this study, we have compared the nodulation phenotype of T3SS mutants of two soybean symbionts—*B. diazoefficiens* USDA110 and *S. fredii* USDA191—on pigeon pea. *B. diazoefficiens* USDA110 and *S. fredii* USDA191 have contrasting nodulation phenotypes on pigeon pea. *B. diazoefficiens* USDA110 produces a large number of ineffective nodules while S. *fredii* USDA191 produces nitrogen-fixing nodules on pigeon pea roots. Interestingly, *B. diazoefficiens* Δ136, a T3SS mutant of *B. diazoefficiens* USDA110, is able to produce nitrogen-fixing nodules on the same host. Previous studies have examined the role of the T3SS on legume nodulation [43]. Ineffective nodulation of some soybean plant introduction lines by *B. diazoefficiens* have been reported earlier [44,45]. As observed in our study, these ineffective nodules were Fix- and lacked leghemoglobin [45]. Krause and her associates examined the nodulation properties of the *B. diazoefficiens* USDA110 and its T3SS mutants, including *B. diazoefficiens* Δ136 used in this present study, on the three different legume hosts, soybean (*Glycine max*), cowpea (*Vigna unguiculata*), and purple bush bean (*Macroptilium atropurpureum*) [21]. In the case of soybean and cowpea, though the T3SS mutants displayed an initial delay in nodulation, the overall nodule number was not significantly different when compared with that of wild type. In contrast, the T3SS mutants produced lower numbers of nodules on bush bean, but these nodules were much larger and revealed a two-fold increase in the nitrogenase activity when compared to the wild type [46]. Unlike the earlier studies, here we have examined the nodulation phenotype of T3SS mutants of two different soybean symbionts on the same host. A summation of the results from all these studies reveal that inactivation of T3SS could lead to different nodulation phenotypes on different legume hosts.

Nodulation outer proteins (Nops) have been shown to function either to promote or suppress nodulation in a strain-cultivar-dependent manner [19,20,47]. USDA257, which produces several Nops in a NodD and flavonoid dependent manner, is unable to nodulate agronomically improved North American cultivars, but forms nitrogen-fixing nodules on primitive soybean cultivars. In contrast, the T3SS mutants of USDA257 forms nitrogen-fixing nodules on all tested soybean cultivars indicating that USDA257 Nops have a detrimental effect on the nodulation of commercial soybean cultivars. In contrast, USDA 191 and *S. fredii* HH103, which also produce almost the same set of Nops as USDA257, are able to form nitrogen-fixing nodules on both the primitive and commercial soybean cultivars. However, we cannot rule out the possibility that both USDA 191 and *S. fredii* HH103 may secrete unique Nops that enable them to nodulate advanced commercial soybean cultivars. In our present study, we report that both USDA 191 and *B. diazoefficiens* USDA110 produce fix+ and fix- nodules on pigeon pea, respectively. Both USDA191 and *B. diazoefficiens* USDA110 secrete Nops in a T3SS manner [19,21]. However, these two rhizobia produce markedly different mixtures of Nops [21,48]. It is possible that the presence or absence of individual Nops or synergistic interactions among them could be responsible for the different nodulation phenotypes of USDA191 and *B. diazoefficiens* USDA110 on pigeon pea. However, other bacterial signals, such as Nod factors, exopolysaccharides (EPS), and lipopolysaccharides (LPS), may also play a role in eliciting Fix+ and Fix- nodules by USDA 191 and *B. diazoefficiens* USDA110 on pigeon pea.

A survey of the literature indicates that pigeon pea can be nodulated by a wide range of rhizobia including Bradyrhizobium and fast-growing rhizobia [49]. However, very little is known about pigeon pea nodule anatomy and ultrastructure. Our study demonstrates that T3SS mutants of *S. fredii* USDA191 and *B. diazoefficiens* USDA110 produce determinate nodules on pigeon pea roots which exhibit ontogeny and histological features characteristic of determinate nodules of other legumes [50]. Ultrastructural investigation by light and electron microscopy revealed the complete absence of the microsymbiont inside nodules initiated by *B. diazoefficiens* USDA110. The formation of ineffective nodules by certain strains of rhizobia on their hosts have been well documented in the literature [45,51,52,53]. For example, *Rhizobium leguminosarum* strain 1019 produces small, round, white nodules on the lateral roots of the garden pea [54]. These ineffective nodules may be similar to or differ from effective nodules of the same species in both their physiology and structure [55,56,57]. Even though the presence of bacterial microsymbiont is essential for formation of nitrogen-fixing root nodules, some legumes can develop empty nodules in the absence of rhizobia [58,59,60,61]. Histological investigations of spontaneous nodules formed on *Lotus japonicus* revealed the absence of infection threads and rhizobia inside these nodules similar to the situation observed in pigeon pea nodules formed by USDA110. Interestingly, the empty nodules were restored into nitrogen-fixing nodules when the T3SS of *B. diazoefficiens* was mutated. This observation suggests that T3SS effectors may also be involved in determining if the rhizobium/legume interaction leads to effective or ineffective nodules.

Ultrastructural investigation of Pigeon pea nodules initiated by *S. fredii* USDA191, *S. fredii* RCB26, and *B. diazoefficiens* Δ136 reveal the accumulation of large amounts of PHB within the bacteroids. Interestingly, PHB synthesis directly competes with N_2_ fixation for reductant and thus accumulation of PHB could lower nitrogen fixation. A previous study has established a negative correlation between the rate of nitrogen fixation and PHB accumulation. For example, a PHB^−^ mutant of *Rhizobium etli* was able to fix significantly more nitrogen than the isogenic PHB^+^ wild type [62]. The physiological role of PHB accumulation in pigeon pea is not clear. Several functional roles for PHB has been proposed [63]. Those include a role in protecting nitrogenase from O_2_-inactivation, providing energy for the differentiation of rhizobia into larger N_2_-fixing bacteroids, improving rhizobia fitness, carbon and energy supply for bacterial reproduction, and survival during starvation. Additional studies using inoculants showing different symbiotic effectiveness will be required to establish a correlation between PHB accumulation and nitrogen fixation in pigeon pea.

It has been reported that pigeon pea is frequently poorly nodulated and that commercial inoculation does not improve crop yields [64]. In such instances, plant growth-promoting rhizobacteria (PGPR) have been used for boosting crop yields. A significant increase in pigeon pea growth and nitrogen fixation was reported when AR-2-2 k (a rhizobium specific for pigeon pea) was used in combination with different rhizobacteria (*Pseudomonas putida*, *P. fluorescens*, or *Bacillus cereus*) as an inoculum for pigeon pea sown seeds [65]. However, due to the semiarid environment, the potential of introducing rhizobial strains to improve pigeon pea productivity and nodule formation is not easily achievable because of the high level of competition with the native rhizobial population. As a consequence of their superior competitiveness, these indigenous bacteria negate the positive effect of commercial inoculants. In our study, we found that inoculation of pigeon pea with a T3SS mutant of *B. diazoefficiens* resulted in nitrogen-fixing nodules. Moreover, the T3SS mutant was able to outcompete the inefficient *B. japonicum* USDA110 for nodule occupancy in coinoculation experiments. The results of our study suggest that use of T3SS mutants of *B. japanicum* as commercial inoculants may be a viable approach to overcome the competitive ability of inefficient indigenous rhizobia.

## 4. Materials and Methods

### 4.1. Bacteria Strains and Growth Condition

*Bradyrhizobium diazoefficiens* USDA110, *Sinorhizobium fredii* USDA191, and T3SS mutant *Sinorhizobium fredii* USDA RCB26 [39] are from our laboratory collections. A T3SS mutant of *B. diazoefficiens* USDA110 Δ136 (lacks a functional *ttsI* gene and several neighboring genes) was obtained from Dr. Michael Göttfert (Technische Universität Dresden). A physical and genetic map of T3SS locus of *S. fredii* USDA191 and *B. diazoefficiens* USDA110 is shown in Figure S3. Rhizobia were grown in a liquid yeast extract mannitol (YEM) medium on a shaker at 30 °C for 3–5 days.

### 4.2. Nodulation Assay

Pigeon pea seeds were surface-sterilized in 50% bleach (2.5% NaClO) and germinated on 1% water agar plates for 5 days in a 30 °C incubator. *S. fredii* USDA 191, *B. diazoefficiens* USDA110, *S. fredii* RCB26, and *B. diazoefficiens* Δ136 were grown in a liquid YEM medium and were harvested by centrifugation at 7700× *g* for 15 min. The cell pellet resulting from this process was resuspended in liquid YEM, to a final concentration of 4.5 × 10^6^ cells/mL. Roots of germinated pigeon pea seedlings were placed in 2 mL Eppendorf tubes containing rhizobia culture, for 60 s. After that, the seedlings were transferred to sterilized Leonard jars containing vermiculite and were placed in a growth chamber. Plants were grown at a constant temperature of 28 °C with a light intensity of 500 μmol of photons per square meter per second, and a 12-h light period. Nodules harvested after 30 DAI (DAI) were used immediately for anatomical studies or stored at −80 °C. The nitrogen fixation rate, measured by acetylene reduction assay, was performed by the method of Schwinghamer et al. [66] on whole root systems containing intact nodules, after removal of the vegetative portion of the plant. Three individual root systems were analyzed for nitrogenase activity for each treatment, over a 25 min time course with air samples taken every 5 min. Activity was collected as the production of ethylene (C_2_H_4_) over time, and calculated to nmol per hour per mg of dry weight of all nodules within the sealed assay vial.

### 4.3. Light and Electron Microscopy Images

Pigeon pea nodules harvested at 30 DAI were embedded in paraffin as described earlier [67]. Paraffin-embedded nodules were sectioned with a microtome to a thickness of 10 μm and were stained with hematoxylin and eosin. For ultrastructural studies, 30 DAI old nodules were cut into 2- to 4-mm pieces with a razor blade and immediately fixed in buffered 2.5% glutaraldehyde (pH 7.2; 50 mM sodium phosphate) at room temperature for 4 h. Then, the tissues were washed four times, at 15-min intervals, with 50 mM phosphate buffer (pH 7.2) and were postfixed with 2% aqueous osmium tetroxide for 1 h at room temperature. After several rinses in distilled water, the samples were dehydrated in a graded acetone series and were infiltrated with Spurr’s resin. Thin sections were cut with a diamond knife and collected on uncoated copper grids. Sections were stained with 0.5% aqueous uranyl acetate and 0.4% aqueous lead citrate and viewed with a JEOL JEM 100B (Tokyo, Japan) electron microscope at 100 kV.

### 4.4. Isolation of Extracellular Proteins

*Sinorhizobium fredii* USDA 191 and *S. fredii* RCB26 were grown in liquid YEM medium either in the absence or presence of apigenin (1 µM) for 3 days at 30 °C. *B. diazoefficiens* USDA110 and *B. diazoefficiens* Δ136 were also similarly grown in liquid YEM medium except the period of growth was extended to 5 days and genistein (1 µM) was used as an inducer. These cultures were harvested by centrifugation at 7700× *g* for 15 min. The supernatant was used for the isolation of extracellular proteins essentially as described previously [68].

### 4.5. One-Dimensional Gel Electrophoresis

For obtaining total nodule protein, 80 mg of 30 DAI nodules were ground into a fine powder using a mortar and pestle. One ml of SDS-PAGE sample buffer containing 2% of β-mercaptoethanol was added to the fine powder and vortexed for 30 min. The slurry was clarified at 16,100× *g* for 10 min. The clear supernatant was removed and boiled for 5 min, and 10-μL aliquots were used for electrophoresis. Nodule proteins and rhizobia extracellular proteins were resolved with 15% gels using a Hoeffer SE 250 mini-Vertical electrophoresis apparatus (GE Healthcare). Separation was achieved with a constant 20 mA/gel and a typical run time of 1.2 h. Gels were removed from the cassette and placed immediately in Coomassie Blue R-250 solution.

### 4.6. 2-D Electrophoresis

For 2-D electrophoretic analysis, 200 mg of nodules were placed into a cold mortar and pestle with a small amount of acid washed sand and 5 mL of 100 mM Tris-Cl, pH 8.8, containing 17% *w*/*v* sucrose, 100 mM NaCl, and 0.4% *v*/*v* β-mercaptoethanol and ProteaseArrest (G-Biosciences, St. Louis MO, USA). The mixture was ground into a liquid, placed into a 15 mL Corning tube, vortexed heavily, and centrifuged at 400× *g* for 10 min at 4 °C to remove plant material. The supernatant was placed into a fresh 15 mL Corning tube and centrifuged at 8000× *g* for 20 min at 4 °C to separate nodule cytosol from bacteroids. The supernatant was placed into a fresh tube, kept on ice, and adjusted to 0.9 M sucrose. An equal volume of phenol (saturated; pH 4.3) was then added and mixed with rotation for 30 min at 25 °C. The phenolic phase was obtained after centrifugation at 5000× *g* for 20 min at 25 °C in a swing-bucket rotor. The upper phenolic phase was removed and added to ten volumes of freshly prepared 100% methanol with 0.1 M ammonium acetate to precipitate proteins. The solution was kept 2 h at −80 °C to promote precipitation, followed by centrifugation at 8000× *g* for 20 min at 4 °C. The supernatant was discarded, and protein was suspended vigorously in a freshly prepared solution of 100% methanol with 0.1 M ammonium acetate containing 0.01 M dithiothreitol. Washing of the insoluble proteins was repeated two more times with the same solution, with incubation at −20 °C for 20 min between centrifugations. Washing of the insoluble proteins was repeated three additional times with a freshly prepared solution of 100% acetone containing 0.01 M DTT with incubation at −20 °C for 20 min followed by centrifugation. Following the final wash, the protein pellet was air dried slightly and dissolved in a small volume of 7 M urea, 2 M thiourea, 1% *w*/*v* CHAPS, and 2% *w*/*v* C7BzO (Sigma, St. Louis MO, USA) using all ultrapure electrophoresis grade reagents.

Protein estimation was performed following the method of Bradford and 300 µg of protein sample was loaded per IEF strip using overnight in-gel passive rehydration. Isoelectric focusing and second dimension SDS-PAGE was carried out as described earlier [69]. Following electrophoresis, the second dimension gels were fixed in 5:4:1 (methanol:water:acetic acid) for 30 min, followed by two brief rinses in water, and stained in a Coomassie G-250 solution overnight. Gels were scanned using an Epson V700 Perfection scanner. Proteome differences were analyzed using Delta2D image analysis software version 4.41(Decodon, Greifswald, Germany).

### 4.7. Western Blot Analysis

Nodule proteins and extracellular proteins were first resolved on 15% SDS-PAGE gels as described earlier. The separated proteins were electrophoretically transferred onto nitrocellulose membranes for 1 h. Following the transfer, the membranes were stained with Ponceau S to visually confirm the transfer. Nitrocellulose membrane were then incubated with 3% dry milk powder and dissolved in Tris-buffered saline (TBS; pH 7.4) for 1 h at room temperature with gentle shaking. After that, the nitrocellulose membrane was incubated with leghemoglobin or nitrogenase antibody that was diluted 1:20,000 in TBS containing 3% dry milk powder. For the detection of Nops, a cocktail of antibodies raised individual Nops were used at a final dilution of 1:10,000. Nonspecific binding was eliminated by washing the membrane three times (10 min for each wash) with TBS containing 0.05% Tween-20 (TBST). Bound antibodies were detected by incubating the nitrocellulose membrane with 1:20,000 of goat anti-rabbit IgG−horseradish peroxidase conjugate chemiluminescent antibody (Bio-Rad, Hercules, CA, USA) for 1 h. Following the incubation, the membrane was washed three times with TBST as mentioned above. Immunoreactive polypeptides were visualized by incubation of the membrane with SuperSignal West Pico enhanced chemiluminescent substrate (ThermoFisher, St. Peters, MO, USA).

### 4.8. Competitive Nodulation Assay

The competitive ability of pigeon pea microsymbionts was evaluated using inoculant mixtures where the proportions of wild type *B. diazoefficiens* and its T3SS mutant varied from 1:10 to 10:1. Roots of five-day-old pigeon pea seedlings were placed in 2 mL Eppendorf tubes containing the desired inoculation mixtures for 60 s. Following this step, the seedlings were transferred to sterilized Leonard jars containing vermiculite and plants were grown in a growth chamber under same conditions described earlier. Nodules were harvested 30 DAI and each nodule was dissected into two halves. These nodules were separated into two groups: (1) *B. diazoefficiens* USDA10 initiated nodules that were small with white interior and (2) Δ136 initiated nodules that were bigger and exhibited a pink interior. A minimum of 100 nodules were evaluated by these criteria to determine the nodule occupants for each of the inoculation mixtures.

### 4.9. Statistical Analysis

One-way ANOVA tests were performed using JMP Version 11 software (SAS Institute, Cary, NC, USA). For any ANOVA tests that showed significant differences at the *p* < 0.05 level, means were then compared using *t*-test ad hoc tests (*α* = 0.05 significance level cutoff).

## Supplementary Materials

Supplementary materials can be found at https://www.mdpi.com/1422-0067/20/5/1091/s1.

## Abbreviations

*NF*	*Nod factor*
T3SS	Type three secretion system
Nops	Nodulation outer proteins
*S. fredii*	*Sinorhizobium fredii*
*B. diazoefficiens*	*Bradyrhizobium diazoefficiens*
TEM	Transmission electron microscopy
PBM	Peribacteroid membrane
DAI	Days after inoculation
PHB	Poly β-hydroxybutyrate
EPS	Exopopolysaccharides
LPS	Lipopolysaccharides
PGPR	Plant growth-promoting rhizobacteria
