# Supplementary material for: Classical Soybean (Glycine max (L.) Merr) Symbionts, Sinorhizobium fredii USDA191 and Bradyrhizobium diazoefficiens USDA110, Reveal Contrasting Symbiotic Phenotype on Pigeon Pea (Cajanus cajan (L.) Millsp)
Source: Int J Mol Sci. 2019 Mar 3;20(5):1091. doi: 10.3390/ijms20051091 (PMC6429105; doi:10.3390/ijms20051091)
Supplement: Supplementary file 1 [file ijms-20-01091-s001.pdf]

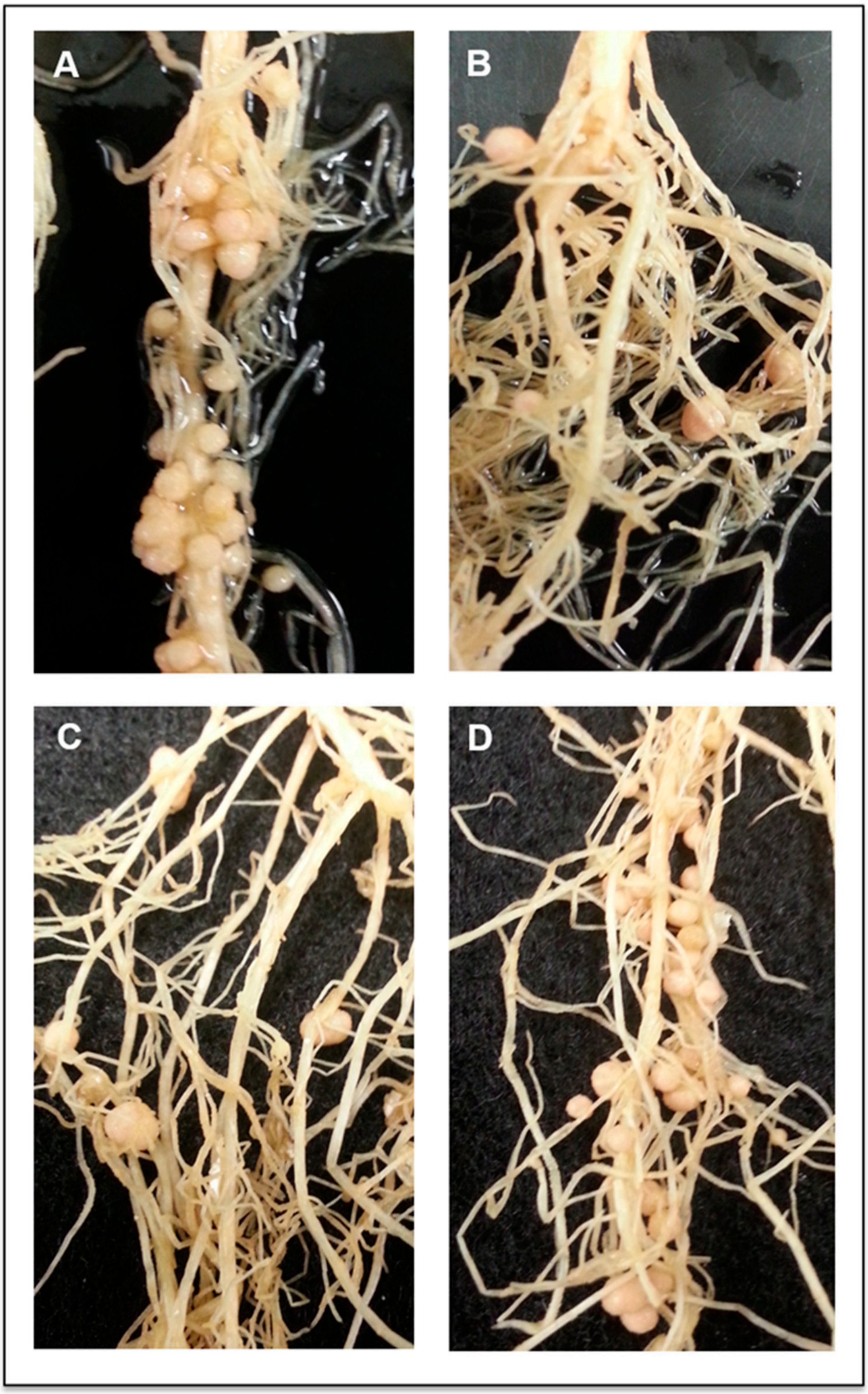

Figure S1. Pigeon pea root nodules. Pigeon pea roots inoculated with *B. diazoefficiens* USDA110 (A), *B. diazoefficiens* Δ136 (B), *S. fredii* USDA191 (C), and *S. fredii* USDA RCB26 (D) were photographed 30 DAI. Note the differences in the nodule size and number initiated by the wild-type and T3SS mutants.

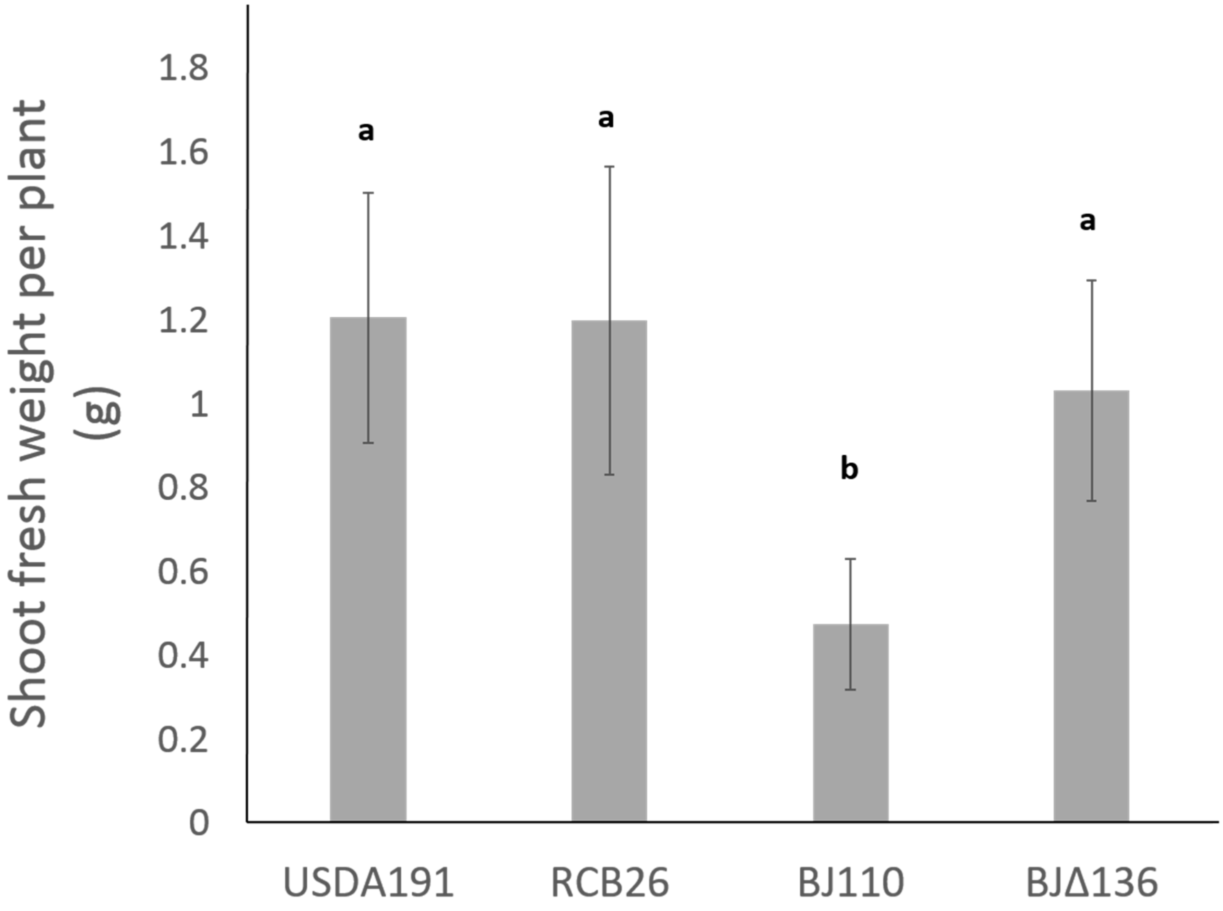

Figure S2. Shoot fresh weight of pigeon pea plants inoculated with *B. diazoefficiens* USDA110, *S. fredii* USDA191 and T3SS mutants. Bars with same letter are not significantly different. Values are presented as mean ± SD (n = 12).

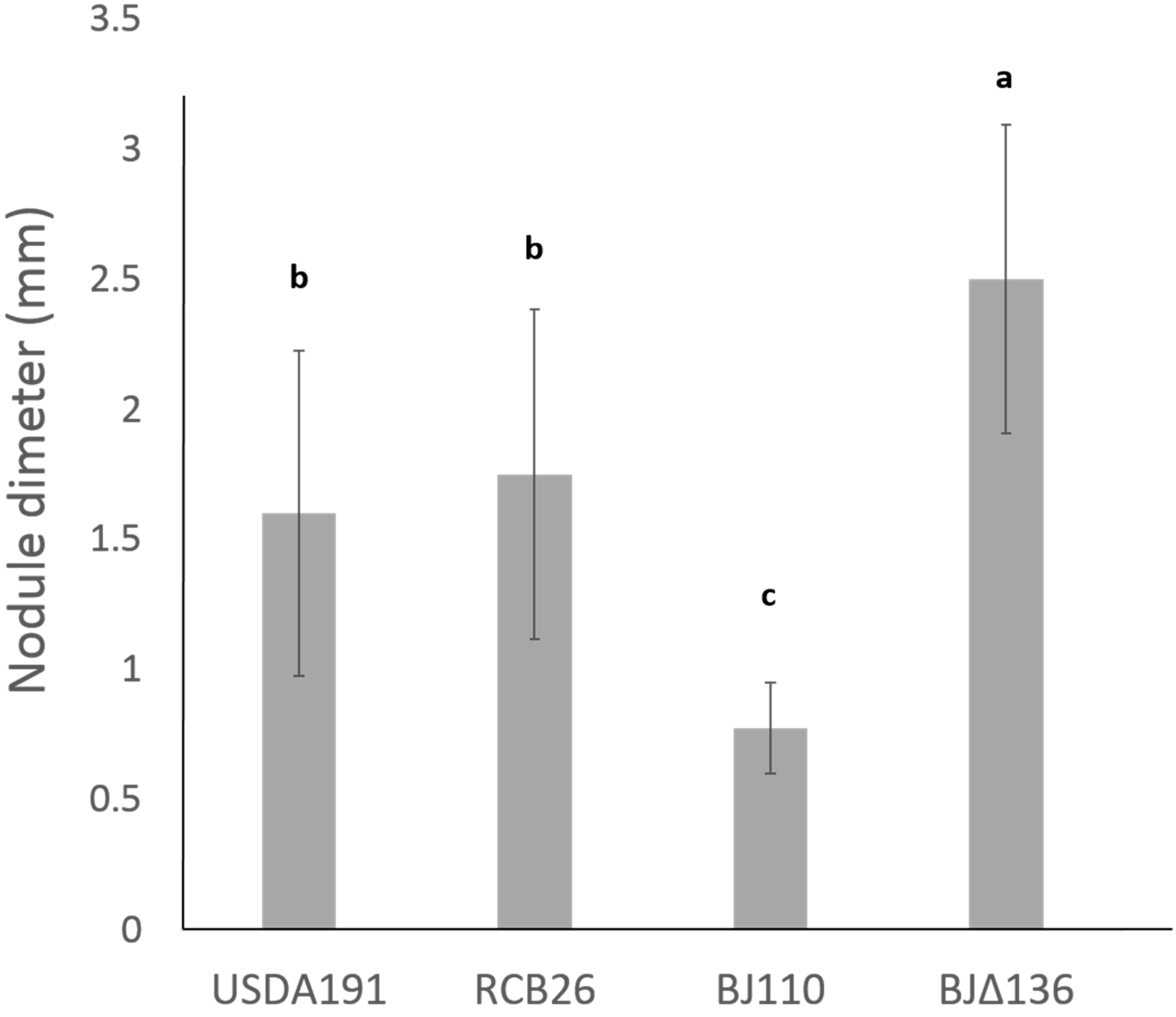

Figure S3. Size of nodules elicited by with *B. diazoefficiens* USDA110, *S. fredii* USDA191 and T3SS mutants on pigeon pea. Bars with same letter are not significantly different. Values are presented as mean ± SD (n = 12).

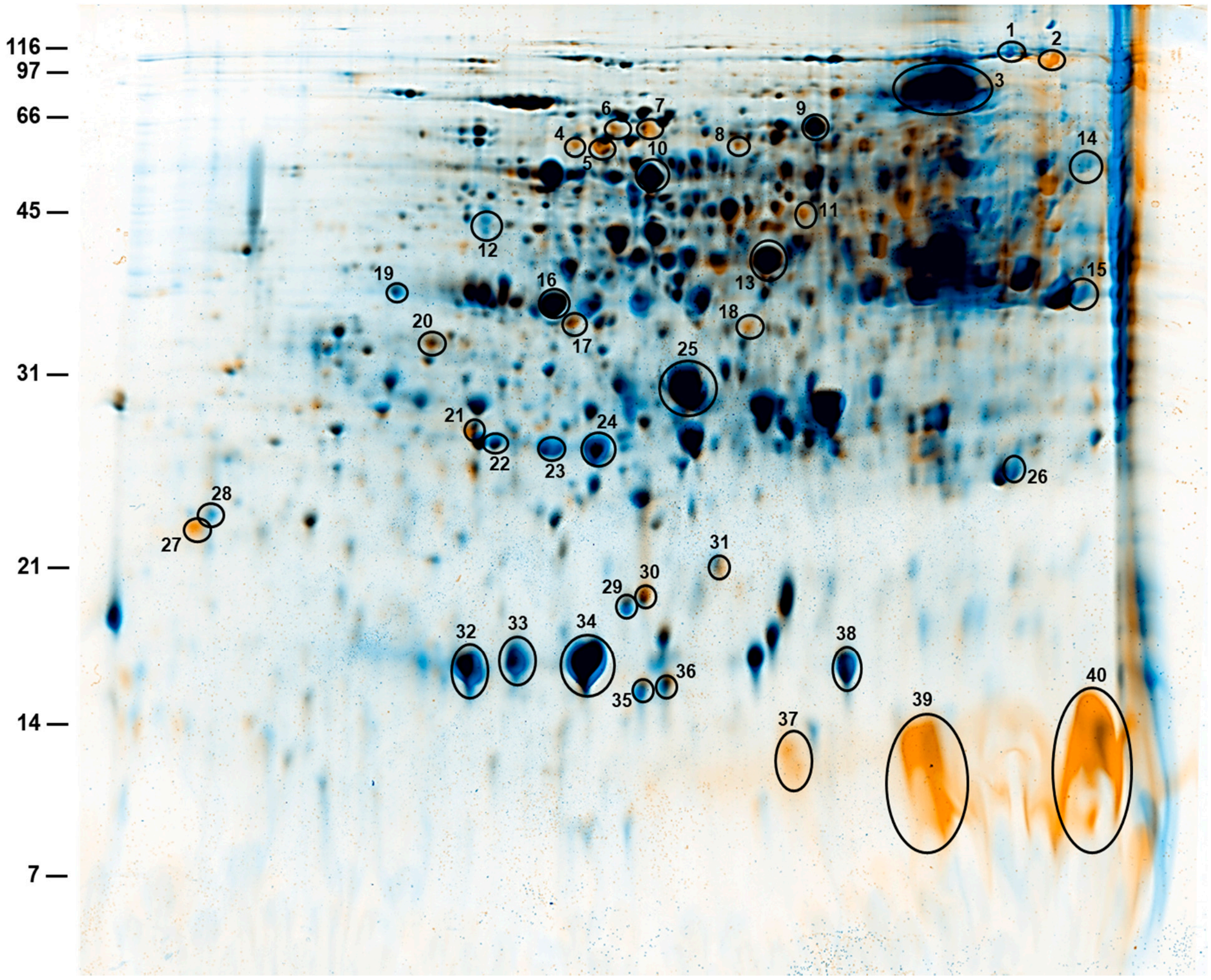

Figure S4. Overlay of two 2D-PAGE of pigeon pea nodule cytosolic proteins. An equal volume of identically extracted proteins from *B. diazoefficienes* USDA110 (blue) and the T3SS mutant *B. diazoefficienes* Δ136 (orange) were separated and visualized as described earlier [69]. Image analysis was done using Decodon Delta2D software to gain normalized % spot volume (differential gel imaging). Individual images were assigned either a blue or orange color. Color differences between the two samples indicates a higher or lower amount on that particular protein in that sample, since proteins of equal amount between samples are shown in black. Software maximizes spot detection and minimizes background. The position and sizes of protein markers in kDa are shown on the left of the figures. Numbered circles correspond to the #'s in Supplemental Table 1.

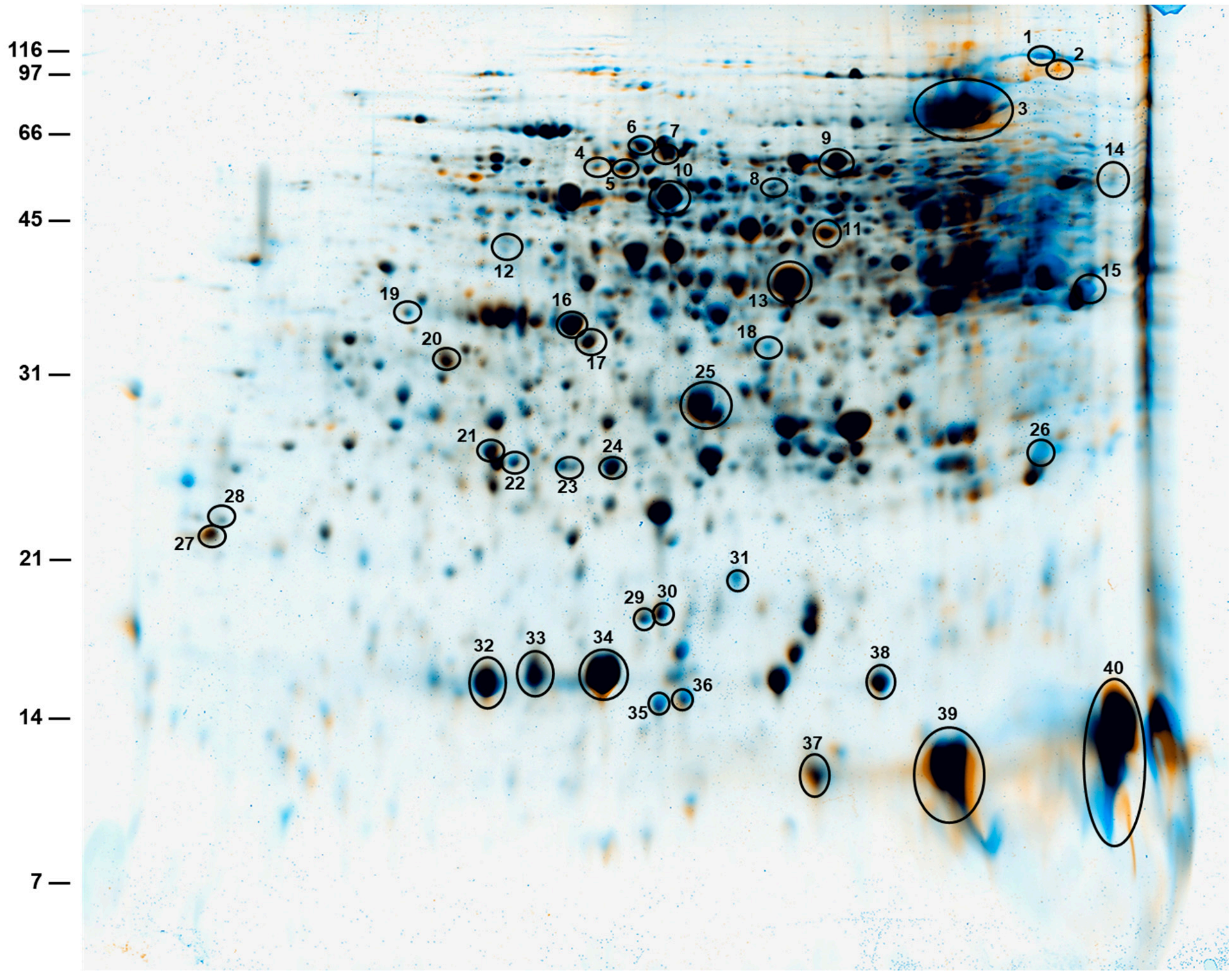

Figure S5. Overlay of two 2D-PAGE of pigeon pea nodule cytosolic proteins. An equal volume of identically extracted proteins from *S. fredii* USDA191 (blue) and the T3SS mutant RCB26 (orange) were separated and visualized as described earlier [69]. Image analysis was performed as described in supplemental figure 4. The position and sizes of protein markers in kDa are shown on the left of the figures. Numbered circles correspond to the #'s in Supplemental Table 2.

Table S1. Spot volume comparison of 40 spots between pigeon pea nodule cytosol from nodules of *B. diazoefficienes* USDA110 and the T3SS mutant *B. diazoefficienes* Δ136. % volume for each spot was obtained as described earlier (Supplemental Figure 4). The ratio of % spot volume between 110 and Δ136 demonstrate the protein differences between those two samples. Values > 2.0, or < 0.5 delineate significant differences between that protein. Spot #'s correspond to those shown in Supplemental Figure 4.

Spot #	% volume 110	% volume Δ136	Ratio Δ136 / 110
1	0.135	0.013	0.095
2	0.006	0.291	52.740
3	3.828	3.340	0.873
4	0.014	0.090	6.572
5	0.055	0.240	4.356
6	0.037	0.115	3.119
7	0.057	0.212	3.710
8	0.011	0.054	4.938
9	0.436	0.412	0.944
10	0.943	0.925	0.981
11	0.005	0.088	18.987
12	0.089	0.027	0.302
13	0.792	1.008	1.272

14	0.088	0.026	0.292
15	0.231	0.081	0.351
16	0.750	0.761	1.014
17	0.052	0.143	2.762
18	0.007	0.093	12.664
19	0.187	0.054	0.289
20	0.125	0.234	1.866
21	0.115	0.211	1.833
22	0.214	0.094	0.438
23	0.544	0.180	0.331
24	0.943	0.378	0.401
25	0.410	0.351	0.855
26	0.305	0.146	0.477
27	0.039	0.265	6.721
28	0.130	0.063	0.483
29	0.229	0.108	0.473
30	0.156	0.329	2.102
31	0.164	0.211	1.286
32	1.770	0.886	0.501
33	1.148	0.530	0.462
34	3.118	2.200	0.706
35	0.256	0.136	0.532
36	0.314	0.269	0.858
37	0.039	0.364	9.300
38	0.955	0.483	0.505
39	0.464	5.305	11.436
40	1.202	9.086	7.559

Table S2. Spot volume comparison of 40 spots between pigeon pea nodule cytosol from nodules of *S. fredii* USDA191 and the T3SS mutant *S. fredii* RCB. % volume for each spot was obtained as described earlier (Supplemental Figure 4). The ratio of % spot volume between 191 and RCB demonstrate the protein differences between those two samples. Values > 2.0, or < 0.5 delineate significant differences between that protein. Spot #'s correspond to those shown in Supplemental Figure 5.

Spot #	% volume 191	% volume RCB	Ratio RCB / 191
1	0.112	0.024	0.217
2	0.023	0.195	8.442
3	1.936	1.531	0.801
4	0.042	0.054	1.289
5	0.149	0.166	1.111
6	0.212	0.177	0.834
7	0.222	0.270	1.208
8	0.080	0.065	0.809
9	0.419	0.445	1.060
10	0.791	0.743	0.939
11	0.139	0.278	2.007
12	0.108	0.072	0.639
13	1.145	1.281	1.118
14	0.046	0.064	1.393
15	0.299	0.104	0.348
16	0.576	0.585	1.017
17	0.211	0.259	1.224
18	0.062	0.022	0.347
19	0.068	0.048	0.710
20	0.230	0.299	1.301
21	0.264	0.292	1.106
22	0.125	0.101	0.806

23	0.199	0.155	0.777
24	0.379	0.346	0.914
25	1.556	1.473	0.959
26	0.308	0.170	0.554
27	0.233	0.376	1.615
28	0.080	0.077	0.960
29	0.162	0.145	0.898
30	0.239	0.212	0.889
31	0.190	0.078	0.409
32	1.226	1.100	0.897
33	0.744	0.693	0.931
34	2.312	2.305	0.997
35	0.245	0.117	0.476
36	0.245	0.172	0.703
37	0.618	0.849	1.374
38	0.487	0.439	0.902
39	3.891	4.779	1.228
40	5.888	6.289	1.068
